# Exploring AI tool adoption in higher education: evidence from a PLS-SEM model integrating multimodal literacy, self-efficacy, and university support

**DOI:** 10.3389/fpsyg.2025.1619391

**Published:** 2025-09-08

**Authors:** Zixuan Zhao, Qi An, Jiaqi Liu

**Affiliations:** ^1^College of Tourism and Landscape Architecture, Guilin University of Technology, Guilin, China; ^2^Endicott College of International Studies, Woosong University, Daejeon, Republic of Korea; ^3^Weifang University of Science and Technology, School of Civil Engineering, Weifang, China

**Keywords:** technology acceptance model, multimodal literacy, self-efficacy, university support, structural equation modeling

## Abstract

**Introduction:**

Framed within the technology acceptance model, this study examines how multimodal literacy, self-efficacy, and university support affect students’ attitudes toward artificial intelligence tools and the students’ intentions to adopt them.

**Methods:**

Survey data from 498 students were analyzed using PLS-SEM 4.0 and SPSS 29.

**Results:**

The findings showed that the perceived usefulness of the AI tools was the strongest predictor of both attitude toward the tools and intention to use them. All three antecedent variables (multimodal literacy, self-efficacy, and university support) significantly impacted perceived usefulness and perceived ease of use.

**Discussion:**

By integrating individual dimensions and also institutional dimensions into the technology acceptance model, this study offers fresh insight into how AI tools might take root more effectively in higher education.

## 1 Introduction

Artificial intelligence (AI) is pushing for a fast transformation in global higher education (Zouhaier, 2023), and it is changing how students learn and how educators teach and evaluate students (Holmes and Porayska-Pomsta, 2023). Several AI technologies have proved that they can help with the acquisition of knowledge and can, among other features, make personalized learning possible by creating custom-made educational experiences (Adiguzel et al., 2023). These tools also make things more accessible (Caldarini et al., 2022) and support the decision-making of academic professionals (Farhi et al., 2023). Indeed, with the acceleration of digital transformation throughout the education sector, the use of AI technologies in education has increased significantly (Viswanath Venkatesh et al., 2003). These smart tools not only involve learners more and help them understand better, they also help students to develop the basic skills needed to prepare for a career (Adiguzel et al., 2023). However, Holmes and Porayska-Pomsta (2023) showed that students demonstrate markedly different levels of acceptance and engagement with AI technologies. Students tend to embrace AI tools for their studies when they find them practical and easy to use for their specific tasks (Ma et al., 2024).

To account for the variability in adoption behaviors, this study employed the technology acceptance model (TAM), which says that how useful people think something is (i.e., its perceived usefulness [PU]) and how easy they think it is to use (i.e., its perceived ease of use [PEOU]) are the main factors that decide how people judge and act toward new technologies (Chen et al., 2024). Multimodal literacy (ML) (Slimi, 2023), self-efficacy (SE) (Fan and Cui, 2024), and university support (US) (Sova et al., 2024) are presumed to affect students’ adoption of AI tools (Yao and Wang, 2024), but the processes by which these relationships occur remain underexplored. That may be because prior TAM-based studies have typically emphasized broad psychological determinants and have paid less attention to the specific learner competencies and institutional contexts relevant to educational technologies. Given the rapidly evolving educational environment and increasing complexity of digital tool adoption, there is a clear need to revisit and extend existing acceptance models to better reflect contemporary higher-education contexts. This research therefore addresses that critical gap by explicitly examining how ML, SE, and US affect students’ acceptance of AI technologies in academic contexts.

This study had two main goals. First, it looked at how these three factors affect how students think about AI tools’ usefulness and ease of use. Then, the study checked how the main TAM factors work together to shape students’ integration of AI tools into their learning activities. To guide the analysis, this research sought to answer the following research questions: (1) To what extent do multimodal literacy, self-efficacy, and university support influence students’ perceived ease of use and perceived usefulness of AI tools in academic contexts? (2) How do perceived ease of use and perceived usefulness shape students’ attitudes toward using AI tools? (3) In what ways do students’ attitudes, perceived ease of use, and perceived usefulness contribute to their behavioral intention to use AI tools in higher-education settings? Because university students are not only primary users but also are potential advocates of AI in education (Fošner, 2024), an in-depth understanding of the drivers of students’ AI adoption behavior can help institutions develop more effective strategies for supporting AI-enhanced learning and promoting meaningful, enduring incorporation of AI technologies within tertiary educational contexts.

## 2 Literature review

### 2.1 Key relationships in the technology acceptance model

The technology acceptance model (Davis, 1989) centers around several critical relationships among core constructs—the perceived usefulness and perceived ease of use of AI, and users’ attitudes and behavioral intentions—that have been extensively tested and validated across diverse technological contexts (Venkatesh and Davis, 2000). Despite its robust predictive capabilities, however, the TAM has faced significant theoretical critiques regarding the simplicity and assumed linearity of these foundational relationships (Bagozzi, 2007; Marangunić and Granić, 2015). For example, Tarhini et al. (2017) noted that the TAM typically presupposes stable, rational user behaviors and neglects emotional, situational, and dynamic psychological factors that might mediate or moderate its core relationships. Furthermore, the assumption of universality in these key relationships overlooks potential variability due to cultural influences, disciplinary contexts, and individual differences, thus raising questions about the generalizability of TAM findings in complex educational settings (Teo and Milutinovic, 2015). Given these limitations, a deeper, contextually sensitive exploration of the TAM’s core relationships is necessary to build a full understanding of technology acceptance behaviors, especially in higher education, where a diverse range of contextual and institutional elements shapes learners’ perceptions. Addressing these contextual influences explicitly could significantly extend the TAM’s explanatory power and practical utility in academic environments.

#### 2.1.1 Perceived ease of use (PEOU) and AI attitude

When users perceive that a technology is easy to understand and work with, this factor is called perceived ease of use (Davis, 1989). According to Venkatesh and Bala (2008), users are more likely to accept technologies that are easy to understand and that work well. In this study, perceived ease of AI use reflects how easy students perceive using AI tools to be (Li et al., 2024) and whether the experience is smooth and not stressful (Fošner, 2024). Previous research has found that systems that are easy to use usually lead to users having more positive attitudes toward them (Fagan et al., 2008) because such systems lower the users’ cognitive load (Karahanna and Straub, 1999) and increase people’s satisfaction with using them (Shroff et al., 2011). Hence, this study proposed the following hypothesis:

*Hypothesis 1 (H1):* Perceived ease of use positively affects students’ attitudes toward AI tools.

#### 2.1.2 Perceived usefulness (PU) and AI attitude

Perceived usefulness means how useful or valuable users think a system is for accomplishing their tasks (Davis, 1989). In this study, students’ perceived usefulness of AI tools was largely characterized by the tools’ potential to boost academic performance (Fagan et al., 2008), simplify learning processes, and enhance task completion (Falebita and Kok, 2024). Several studies have indicated that users’ attitudes toward technology adoption are strongly influenced by perceived usefulness (Aljarrah et al., 2016), and offering additional perceived benefits usually leads to a higher level of acceptance (Toros et al., 2024). When students believe that AI gives clear academic benefits, they are likelier to have a more positive attitude (Farhi et al., 2023) and a stronger intention to use it (Slimi, 2023). Thus, the following hypothesis was proposed:

*Hypothesis 2 (H2):* Perceived usefulness positively affects students’ attitudes toward AI tools.

#### 2.1.3 AI attitude and AI intention

Artificial intelligence attitudes are the positive and negative opinions that users form about AI tools on the basis of their interactions and experiences with them (Ajzen and Fishbein, 2000). These attitudes encompass the users’ thoughts, emotional responses, and behavioral inclinations during interactions with AI (Slimi, 2023). Attitude is thought to be a basic psychological thing that affects people’s intention to adopt and keep using new technologies (Kim et al., 2024). A positive attitude is seen as an important factor that influences people to accept new technologies (Ayanwale et al., 2024) and prepares users to use these tools in their daily learning and work tasks (Toros et al., 2024). Prior studies also have suggested that individuals with more favorable attitudes toward AI are generally more willing to adopt such tools (Chen et al., 2024; Teo and Zhou, 2014). Therefore, this study proposed the following hypothesis:

*Hypothesis 3 (H3):* Students’ AI attitudes positively affect their intention to use AI tools.

#### 2.1.4 Perceived ease of use (PEOU) and AI intention

Perceived ease of use (PEOU) affects students’ adoption decisions, particularly concerning emerging AI-based tools and platforms (Venkatesh and Davis, 2000). Users are more inclined to adopt and continue using technologies that have simple interfaces, because the reduced complexity lowers the mental effort required for learning (Yi and Hwang, 2003). Recent research has further underscored PEOU as a critical determinant of user acceptance across educational settings that involve AI-driven tools.

In particular, Rafique et al. (2020) provided empirical support that PEOU significantly predicted students’ behavioral intentions toward mobile library applications, indicating that ease of use was a critical factor influencing user acceptance in digital learning environments. Salar and Hamutoglu (2022) underscored the notion that perceived ease of use plays a predictive role in users’ willingness to engage with cloud computing systems. Similarly, Al-Maroof et al. (2020) demonstrated through an extended technology acceptance model that PEOU directly influenced users’ behavioral intentions to use machine translation tools such as Google Translate, further reinforcing the role of user-perceived ease of use in driving AI adoption. Falebita and Kok (2024) found that PEOU significantly shaped undergraduate students’ intentions in adopting AI technologies, highlighting that user-friendly and intuitive interfaces positively affected acceptance and sustained use. In addition, Al-Adwan et al. (2023) reported a notable link between PEOU and students’ intention to adopt metaverse-based learning platforms, noting variations depending on technological complexity and individual self-efficacy levels.

In that light, given the robust evidence highlighting the impact of PEOU in shaping technology adoption intentions across various studies, particularly in educational settings involving AI tools, this study proposed the following hypothesis:

*Hypothesis 4 (H4):* Perceived ease of use positively affects students’ intention to use AI tools.

#### 2.1.5 Perceived usefulness (PU) and AI intention

Perceived usefulness is a factor that influences user attitudes and directly affects technology adoption behaviors (Venkatesh and Davis, 2000). When users find a technology to be helpful in achieving their goals, they tend to use it more frequently (Adelana et al., 2024; Das and Madhusudan, 2024). Recognized for its impact on user adoption decisions, perceived usefulness has become a core factor in recent research (Yao and Wang, 2024), especially in environments such as online learning platforms (Al-Maroof et al., 2020), Massive Open Online Courses (MOOCs) (Alraimi et al., 2015), educational technologies, and AI-driven applications (Nikolopoulou et al., 2020). Specifically, Alraimi et al. (2015) found that PU was a strong driver of learners’ continuance intention in MOOCs, and that drive was particularly shaped by perceived openness and institutional reputation. Nikolopoulou et al. (2020) provided empirical evidence supporting PU as a key factor in mobile learning acceptance among university students. Both Yao and Wang (2024) and Al-Maroof et al. (2020) demonstrated that perceived usefulness had a significant and direct influence on pre-service teachers’ intention to adopt AI tools, across contexts such as special education and intelligent tutoring systems in biology. Saravanos et al. (2022) explored the adoption of digital educational technologies within an extended TAM3 framework and confirmed that perceived usefulness directly predicted university students’ behavioral intentions toward adopting emerging technologies, including AI-enhanced learning environments. Their findings highlighted that perceived usefulness was consistently influential across multiple contexts, such as interactive AI-driven learning tools and virtual learning platforms, thereby aligning closely with this study’s emphasis on AI adoption in higher education. Accordingly, this study proposed the following hypothesis:

*Hypothesis 5 (H5):* Perceived usefulness positively affects students’ intention to use AI tools.

### 2.2 Multimodal literacy (ML)

In the digital age, relying on reading and writing skills is no longer enough. Multimodal literacy has now become crucial for interpreting, analyzing, and conveying information with the help of AI tools. Emerging from social semiotics (Jewitt, 2008), ML involves the ability to understand and express ideas (Kress, 2010) through various forms of communication (Walsh, 2010). Multimodal literacy combines forms such as text and images along with sounds and gestures to help people understand and interact with content more effectively. As Kress (2010) observed, multimodal literacy enhances meaning by combining the analysis of sounds, images and text, which reflects the diversity of current digital communication (Bulut et al., 2015). The increasing use of visuals and interactive tools (Bezemer and Kress, 2008) in education highlights their influence on customizing the educational experience and transforming how information is learned (De Oliveira, 2009). In addition, multimodal literacy promotes flexibility (Kress and Selander, 2012) by allowing students to adjust their communication methods on a situational basis and encourages creativity in environments (Rowsell and Walsh, 2011). However, assessing multimodal literacy across disciplines presents challenges: Tan et al. (2020) noted that educators often rely on the traditional rubrics aligned with writing, thus undervaluing multimodal performances and lacking consistent, discipline-sensitive criteria.

A growing trend shows that people with greater levels of multimodal literacy are more engaged cognitively (Gee, 2014) and more adept at using digital technologies effectively (Rapp et al., 2007) for understanding intricate information (Jewitt, 2008) and tackling problem-solving tasks (Ng, 2012). Lu et al. (2024) indicated that people who possess stronger multimodal literacy are more open to using platforms with complex visual content. As Mayer (2009) emphasized, those with stronger multimodal skills are usually better at processing and remembering multimedia content. This process often leads students to perceive digital tools as more intuitive and accessible (Kress, 2010), thereby enhancing their perceived ease of use. Moreover, students with high levels of multimodal literacy often report experiencing less cognitive strain when interacting with complex interfaces, and that in turn lowers their psychological barriers to adoption (Paas and Sweller, 2012).

Indeed, multimodal literacy not only helps users navigate systems more easily, it also enhances perceived usefulness by enabling users to derive valuable information from complex multimodal sources (Lu et al., 2024). Because many AI systems present content through visuals, sound, and interactive elements (Seufert, 2018), students with stronger multimodal literacy skills are better able to interpret and apply the content they encounter. In AI-supported learning environments, this ability allows them to apply multimedia resources more effectively (Moreno and Mayer, 2007), thereby enhancing their perceived value of AI tools. Consequently, this study proposed the following hypotheses:

*Hypothesis 6 (H6):* Multimodal literacy positively affects perceived ease of use of AI tools.

*Hypothesis 7 (H7):* Multimodal literacy positively affects perceived usefulness of AI tools.

### 2.3 Self-efficacy (SE)

Bandura (1986) defined self-efficacy as individuals’ confidence in their ability to take the actions necessary to achieve specific goals. Self-efficacy also involves a sense of assurance in handling tasks successfully (Sáinz and Eccles, 2012) and overcoming obstacles (Wang and Wang, 2024). Zimmerman (2000) argued that highly self-efficacious individuals typically demonstrate persistence and show stronger resilience when facing challenges, and they are more committed to problem-solving (Serap Kurbanoglu et al., 2006), traits closely linked to better academic performance (Wang and Li, 2024). Moreover, self-efficacy is not limited to prior experiences, it also influences how individuals approach future obstacles (Huang et al., 2024). Scholars have increasingly emphasized the important function of self-efficacy in academic contexts (Fathi et al., 2021). For instance, Beile and Boote (2004) reported a clear positive link between self-efficacy and how well students do academically (Berweger et al., 2022). Similarly, Wang et al. (2024) showed that students possessing high self-efficacy displayed increased resilience when confronted with difficulties. Other studies have suggested that self-efficacy also promotes students’ cognitive engagement (Girasoli and Hannafin, 2008) and helps them solve problems more efficiently (Hoffman and Spatariu, 2008).

Prior studies have explored the association between perceived ease of use and perceived usefulness and self-efficacy (Marakas et al., 1998). Pan (2020) demonstrated that college students who were more confident in using tech were more open to trying AI tools, Liwanag and Galicia (2023) emphasized AI’s role in promoting self-directed learning, and Masry-Herzallah and Watted (2024) highlighted the positive effect of AI on engaging with online platforms. Confident users are typically less anxious than their counterparts (Igbaria, 1995) because they tend to stay curious and are good at solving problems independently (Holden and Rada, 2011), and they often find AI technologies approachable and helpful, which enables them to unlock the technologies’ full potential (Agarwal and Karahanna, 2000). Conversely, a lack of self-efficacy frequently creates fear and anxiety about failure, which then prevents the individual from adopting a technology (Moos and Azevedo, 2009). The following hypotheses were consequently proposed:

*Hypothesis 8 (H8):* Self-efficacy positively affects perceived ease of use of AI tools.

*Hypothesis 9 (H9):* Self-efficacy positively affects perceived usefulness of AI tools.

### 2.4 University support (US)

When universities offer reliable infrastructure and clear guidelines, students tend to engage more actively and meaningfully with new technologies such as AI (Claro et al., 2018). This support often takes the form of AI training programs (Brown et al., 2010), curricula that reflect technological advances, and a supportive environment that encourages an exploration of these tools (Hammond et al., 2011). Research increasingly has emphasized the positive outcomes brought about by such initiatives (Cho and Yu, 2015). For instance, AI applications have been linked to more personalized learning experiences (D’Mello et al., 2012), greater learner motivation (Deng and Yu, 2023), and improved academic performance (Winkler and Soellner, 2018). In addition to providing infrastructure, universities also shape students’ ethical and effective use of AI through guidance and structured training programs (Denecke et al., 2023; Sova et al., 2024). Jeilani and Abubakar (2025) demonstrated that perceived institutional support significantly enhanced students’ technology self-efficacy and positively influenced their perceptions toward AI-assisted learning. University support was especially beneficial for students who initially exhibited lower self-efficacy, thus highlighting its crucial role in facilitating their adoption of technology.

Embedding AI into courses (Slimi, 2023) can help students understand its relevance to academic achievements (Chiu, 2024) and future careers (Stutz et al., 2023). In addition to offering courses related to AI, universities can also contribute by providing technical assistance, which not only lowers the barriers to using AI tools (Mori, 2000) but also helps students grow into confident and capable users throughout the process (Zhao et al., 2022). Moreover, evidence from De Oliveira (2024) showed that embedding multimodal literacy training within institutionally supported courses significantly enhanced students’ proficiency with multimodal resources, emphasizing that institutional backing directly amplifies the personal competencies that are essential for technology adoption.

In essence, strong university support has been shown to enhance perceived usefulness by emphasizing academic and career benefits while also contributing to improved perceived ease of use (Williams, 2025). Consequently, this study proposed the following hypotheses:

*Hypothesis 10 (H10):* University support positively affects perceived ease of use of AI tools.

*Hypothesis 11 (H11):* University support positively affects perceived usefulness of AI tools.

**Figure 1:**
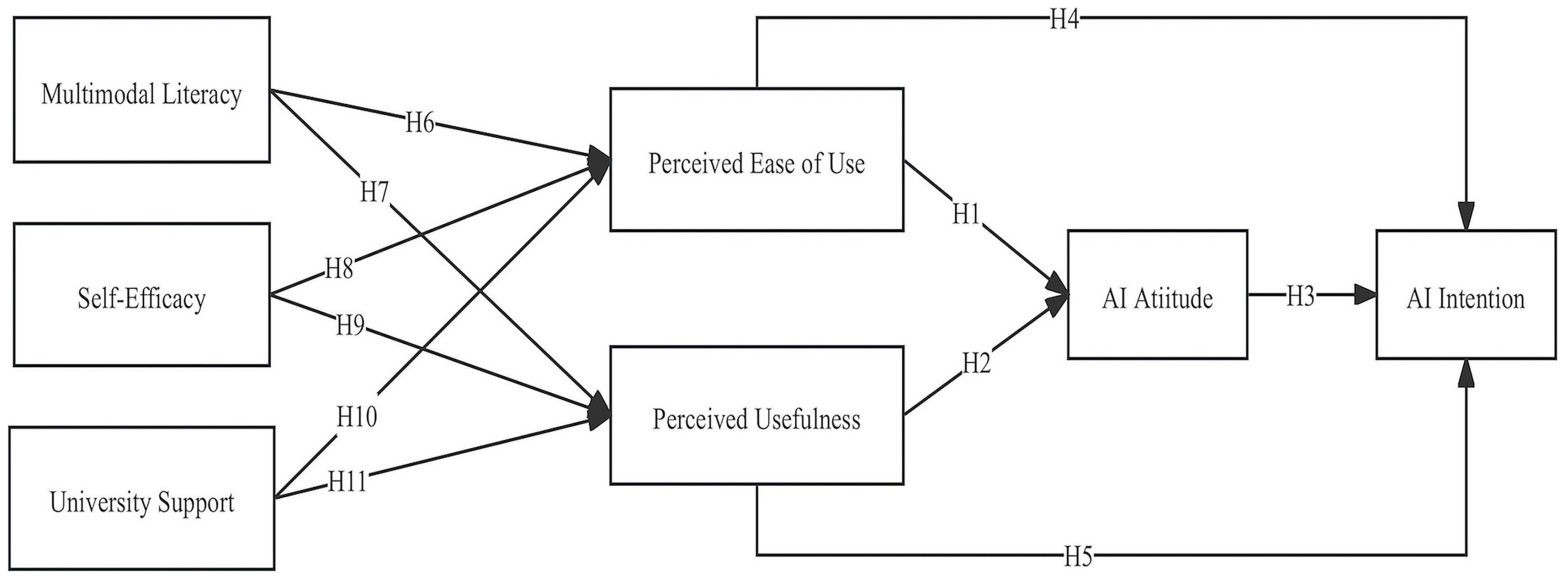
Conceptual model.

Building on insights from prior TAM research, this study began by developing a conceptual model (see Figure 1).

## 3 Research methodology

### 3.1 Research design

Grounded in the TAM, this study investigated students’ adoption of AI tools through a quantitative cross-sectional approach (Bryman, 2016). As Creswell (2009) pointed out, this method effectively supports the analysis of hypothesized relationships. To capture relevant data, a questionnaire was developed to assess the core TAM constructs (PEOU, PU, attitude, intention) and the influencing factors (ML, SE, US). After completion of the data-collection stage, SPSS 29 and SmartPLS 4 were employed to analyze the data. We relied on exploratory factor analysis (EFA) to reduce the risk of common method bias (Iacobucci, 2010), and we verified the variance inflation factor (VIF) values. Subsequently, we assessed internal consistency and construct validity through Cronbach’s alpha, composite reliability, and average variance extracted (AVE). To examine discriminant validity, the study followed Hair et al. (2017) and applied both the Fornell–Larcker criterion and the heterotrait-monotrait (HTMT) ratio test methods. In addition, we employed partial least squares-structural equation modeling (PLS-SEM) to examine the relationships among the constructs and evaluate the proposed hypotheses, because the technique is well suited to exploratory research and handles latent variables effectively (Sova et al., 2024). This study further explored its model fit through key indicators such as path coefficients and *R*^2^ values (Al-Abdullatif and Alsubaie, 2024). To determine the significance of the hypothesized paths, we performed bootstrapping with 5,000 resamples, following the procedure in Hair (2019).

### 3.2 Research instruments

On the basis of the mature tools from previous studies, we developed a questionnaire to ensure reliability and practical relevance, and we ultimately created a tool consisting of nine sections. The first section collected demographic information to provide a clear profile of the research sample (Li et al., 2024). The subsequent seven sections assessed the core constructs of our theoretical model. Each construct was measured using multiple items, rated by participants on a five-point Likert scale ranging from “strongly disagree” to “strongly agree.” Specifically, multimodal literacy was evaluated through 11 items sourced from Bulut et al. (2015), self-efficacy with five items drawn from Falebita and Kok (2024), and university support with three items based on Sykes et al. (2009). For the TAM constructs, we drew four items from Falebita and Kok (2024) to assess perceived ease of use and three items to measure AI attitude. Perceived usefulness and AI intention were assessed with four and three items, respectively, derived from Ayanwale and Molefi (2024). These items have demonstrated high degrees of reliability and validity in prior research, lending support to the measurement instrument adopted for the current study. We also included an open-ended question to gather qualitative insights.

After translating the original English questionnaire into Chinese, two bilingual professionals validated the translation’s clarity. Following Behling and Law (2000), we conducted a back-translation to ensure accuracy and then carefully addressed any discrepancies. Prior to formal distribution of the main questionnaire, a pilot study was carried out with 238 university students to evaluate item clarity. On the basis of participant feedback, minor wording revisions were made.

### 3.3 Ethical considerations

This study abided by ethical standards, with informed consent obtained after delivery of a clear explanation of the study’s aims and its voluntary nature. Participants’ anonymity was respected, and all data were kept confidential. The findings were used solely for academic purposes and presented truthfully, ensuring both transparency and research integrity.

### 3.4 Population and sampling

The population was university students across various disciplines and degree levels who had experienced a past exposure to engaging with AI tools in academic settings. This group was chosen because students are often early adopters of educational technology and are increasingly exposed to AI-driven tools that shape their learning experiences (Selwyn, 2022; Teo, 2011). Participants were recruited from universities nationwide in China, including undergraduates and postgraduates, to capture diversity in educational levels and fields. Data collection was achieved using a non-probability convenience sampling method (Etikan, 2016). Although convenience sampling can limit generalizability, it is widely accepted in research on exploratory technology adoption that requires rapid large-scale data collection (Evans and Mathur, 2005). In total, 498 valid responses were included in the analysis, thereby ensuring sufficient power for statistical testing (Sax et al., 2003).

### 3.5 Data collection methods

The questionnaire was distributed online through Sojump.com from February to March 2025, and the link was distributed through multiple outlets (e.g., WeChat, QQ, in-class QR codes, and the Xiaohongshu social platform) to reach a broad range of students. To encourage more people to complete the survey, the study included a small monetary incentive in the form of a WeChat “red envelope.” This multi-channel distribution strategy and incentive helped improve the response rate and the diversity of respondents (Dillman et al., 2014). To ensure data quality and prevent duplicate submissions, a set of technical safeguards was implemented before launch. A verification step was enabled to block automated or bot-based responses, and IP-level restrictions were applied to ensure that each participant could only submit the questionnaire and receive the reward once. Distribution of the survey was limited to group-based social media platforms, and Sojump’s backend system did not collect any identifying information about participants (e.g., names, phone numbers, or user IDs), thereby ensuring complete anonymity. Altogether, 669 responses were collected. After data cleaning (removing incomplete submissions and those with all identical answers, extremely short completion times, or failed attention-check questions), 498 responses remained for analysis.

### 3.6 Pilot study

A trial study was first implemented with 238 university students to evaluate and refine the questionnaire before the main study. Thomander and Krosnick (2024) emphasized that pilot studies are essential for refining instruments and ensuring their clarity, validity, and reliability. The initial questionnaire contained 51 items (including demographic questions, an attention check item, an open-ended question, and 44 items covering the seven theoretical constructs).

Because some measurement items were adapted to the AI-in-education context, an exploratory factor analysis (EFA) was first carried out to identify the underlying dimensions, verify construct unidimensionality, and refine the scale. This EFA-to-PLS-SEM sequence followed the methodological recommendations outlined by Hair et al. (2022) for scale development and validation.

On the basis of the pilot feedback and analysis, several modifications ultimately were made. Notably, a “Preferring Multimodal Structures” subdimension of the ML scale (five items) was removed due to its subjective nature and misalignment with the TAM’s focus on user behavior. This change improved the conceptual clarity of the ML construct. In addition, any survey items with low factor loadings (below 0.70), high multicollinearity (VIF > 3.3), or poor discriminant validity in the pilot phase were removed to enhance construct quality (Comrey and Lee, 1992; Hair, 2019; Henseler et al., 2015; Kock, 2015; Tabachnick and Fidell, 2013). These revisions resulted in a final instrument of 39 items for the subsequent studies.

### 3.7 Main study

The finalized questionnaire was deployed as described above, and 498 valid responses were retained for analysis. The comprehensive analysis outlined in section 3.1 was applied to this dataset to assess the conceptual framework. Hoyle (2012) noted that PLS-SEM enables researchers to examine multiple relationships simultaneously while controlling for measurement error. It should be noted that, as with any self-reported survey, there was a risk of common method bias (CMB) or social desirability effects, although statistical procedures indicated these were not significant issues. In addition, the sample had a higher concentration of students from eastern and central China, which is acknowledged in the Limitations section as a consideration with regard to generalizability.

## 4 Results

### 4.1 Descriptive statistics

Among the 498 valid participants, 60.2% were females and 39.8% were males. The vast majority (89.8%) were undergraduate students (most in their second or third year), with 10.2% being graduate students. The sample was predominantly drawn from science-technology-engineering-math (STEM)-related majors: Engineering (27.9%) and Medicine (23.5%) together accounted for about half of the respondents, followed by students from the Natural Sciences (15.3%). Non-STEM fields had smaller representations (e.g., Management 14.3%, Economics 6.2%, Arts 3.2%, Law 3.0%, and others were below 3%). The demographic characteristics of the sample are shown in detail in Table 1.

**Table 1:** Participant demographics.

Characteristic		Frequency	Percent
Gender	Male	198	39.8
	Female	300	60.2
Year of study	Freshman	56	11.2
	Sophomore	151	30.3
	Junior	191	38.4
	Senior	49	9.8
	Graduate student	51	10.2
Major	Philosophy	8	1.6
	Economics	31	6.2
	Law	15	3.0
	Education	14	2.8
	Literature	7	1.4
	Natural sciences	76	15.3
	Engineering	139	27.9
	Agronomy	2	0.4
	Medicine	117	23.5
	Management	71	14.3
	Arts	16	3.2
	Military science	2	0.4

As is shown in Table 2, regarding their frequency of AI tool usage, approximately 79.1% of the students stated that they used AI tools at least occasionally, with 42.2% using them often and 36.9% sometimes, while only 1.2% had never used such tools. These data indicated that AI tools are now embedded in students’ digital routines. As Crompton and Burke (2023) noted, this reflects a global trend of widespread AI integration across contexts.

**Table 2:** Patterns and extent of AI tool use in academic work.

Details of use	Extent	Frequency	Percent
AI tool use intensity	Never	6	1.2
	Rarely	53	10.6
	Sometimes	184	36.9
	Often	209	42.2
	Very often	46	9.2
Degree of AI tool use in academic work	Fully AI-generated, submitted without editing	21	4.2
	Mostly AI-generated, edited as needed	173	34.7
	Partially AI-generated, mainly self-completed	263	52.8
	No AI tools used in academic work	41	8.2

Regarding how extensively the students relied on AI-generated content in their academic work, 52.8% indicated that they used AI for partial content generation while completing the majority of their work independently, 34.7% used AI to generate most content but then edited it themselves, and only 4.2% submitted fully AI-generated content without modification. Meanwhile, 8.2% reported not using AI in their academic work at all. These results suggest that the utilization of AI technologies is widespread among students, but those technologies are primarily used as auxiliary tools rather than replacements for conventional tasks. Therefore, this disparity underlines the necessity for institutions to enhance students’ AI-related competencies through targeted training in AI skills (Zhai et al., 2021).

### 4.2 Common method bias

We followed the guidelines of Podsakoff and Organ (1986) and conducted a single-factor test to assess the presence of common method bias (CMB). Exploratory factor analysis revealed a multidimensional structure, with the first factor alone contributing 42.142% to the total explained variance (see Table 3). Because this value falls short of the widely recognized 50% threshold (Hew et al., 2018), we do not consider CMB to be a major concern in the present study.

**Table 3:** Harman’s single-factor test.

Component	% of variance	Cumulative %
1	42.142	42.142
2	9.372	51.514
3	5.560	57.075
4	4.144	61.219
5	3.378	64.597

To further validate the absence of CMB, a VIF analysis was performed (Kock, 2015). Table 4 shows that all VIF values remained well within the acceptable range, spanning from 1.346 to 2.107 and staying under the recommended maximum of 3.3. Therefore, the combined results of the Harman’s test and VIF analysis confirmed that CMB did not materially influence the study’s results.

**Table 4:** VIF results for multicollinearity diagnosis.

Variable	ATT	IN	ML	PEOU	PU	SE	US
ATT		1.915					
IN							
ML				2.107	2.107		
PEOU	2.032	2.098					
PU	2.032	2.603					
SE				1.962	1.962		
US				1.346	1.346		

### 4.3 Measurement model

The results of the tests just described confirmed that the measurement model met the criteria for both reliability and validity. Table 5 presents evidence supporting both internal consistency and convergent validity. Cronbach’s alphas and composite reliability values consistently exceeded 0.80 (Fornell and Larcker, 1981), while each construct’s AVE surpassed the 0.50 threshold (Hair et al., 2012) and all indicator loadings exceeded 0.70 with statistical significance (Hair, 2019). Discriminant validity was supported through both criteria: Table 6 shows that the square root of AVE for each construct exceeded its inter-construct correlations (Sarstedt et al., 2014), while Table 7 reports HTMT ratios that were consistently below 0.85 (Henseler et al., 2015). Together, these findings provide robust support for the model’s convergent and discriminant validity.

**Table 5:** Reliability and validity results.

Items	Outer loadings	α	C.R.	AVE
ATT1	0.882	0.849	0.908	0.768
ATT2	0.855			
ATT3	0.891			
IN3	0.869	0.818	0.892	0.733
IN4	0.850			
IN5	0.848			
ML_EOUMS2	0.719	0.921	0.933	0.560
ML_EOUMS3	0.739			
ML_EOUMS4	0.748			
ML_EOUMS5	0.756			
ML_ICPMS1	0.708			
ML_ICPMS2	0.720			
ML_ICPMS3	0.733			
ML_ICPMS4	0.773			
ML_ICPMS5	0.803			
ML_ICPMS6	0.803			
ML_ICPMS7	0.725			
PEOU1	0.829	0.877	0.916	0.731
PEOU2	0.887			
PEOU3	0.879			
PEOU4	0.824			
PU1	0.846	0.862	0.906	0.707
PU2	0.809			
PU3	0.873			
PU4	0.834			
SE1	0.818	0.880	0.913	0.676
SE2	0.846			
SE3	0.839			
SE4	0.804			
SE5	0.804			
US1	0.881	0.882	0.927	0.809
US2	0.923			
US4	0.894			

**Table 6:** Fornell–Larcker criterion.

Variable	ATT	IN	ML	PEOU	PU	SE	US
ATT	0.876						
IN	0.707	0.856					
ML	0.421	0.443	0.749				
PEOU	0.575	0.615	0.565	0.855			
PU	0.679	0.687	0.539	0.713	0.841		
SE	0.486	0.520	0.693	0.621	0.566	0.822	
US	0.314	0.357	0.491	0.531	0.431	0.430	0.899

**Table 7:** HTMT criterion.

Variable	ATT	IN	ML	PEOU	PU	SE	US
ATT							
IN	0.848						
ML	0.471	0.506					
PEOU	0.665	0.725	0.626				
PU	0.792	0.816	0.600	0.820			
SE	0.562	0.613	0.765	0.706	0.648		
US	0.364	0.421	0.546	0.601	0.494	0.489	

### 4.4 Structural equation modeling (SEM)

As evidenced by the data in Table 8, the structural model outcomes provided support for all of the hypothesized relationships (H1 through H11), with most paths reaching statistical significance. The students’ attitudes toward AI tools (ATT) markedly influenced their AI usage intention (IN) (*β* = 0.416, *p* < 0.001), thus confirming H3. Moreover, PEOU exerted a significant positive effect on the students’ intention to use AI tools (IN) (*β* = 0.177, *p* < 0.001), providing empirical support for H4. Likewise, PU directly predicted intention to use AI tools (IN) (*β* = 0.278, *p* < 0.001), providing solid empirical support for H5.

**Table 8:** Structural model analysis.

Influence	β	SE	*t*-value	*p*-value
ATT → IN	0.416	0.051	8.112	0.000
ML → PEOU	0.152	0.057	2.678	0.007
ML → PU	0.215	0.069	3.138	0.002
PEOU → ATT	0.186	0.051	3.668	0.000
PEOU → IN	0.177	0.045	3.904	0.000
PU → ATT	0.546	0.050	10.871	0.000
PU → IN	0.278	0.056	4.931	0.000
SE → PEOU	0.392	0.054	7.298	0.000
SE → PU	0.340	0.060	5.663	0.000
US → PEOU	0.288	0.048	6.020	0.000
US → PU	0.179	0.050	3.591	0.000

The findings further confirmed that PU had a strong positive influence on AI attitude (ATT) (*β* = 0.546, *p* < 0.001), thus lending support to H2. Similarly, H1 was supported because the path from PEOU to AI attitude (ATT) also was both significant and positive (*β* = 0.186, *p* < 0.001). The data demonstrate that both perceived ease of use and perceived usefulness contributed to students’ attitudes toward AI tools, although the influence of usefulness was the more pronounced of the two.

Multimodal literacy positively influenced both PEOU (*β* = 0.152, *p* = 0.007) and PU (*β* = 0.215, *p* = 0.002). While the effect sizes were relatively small, the findings nonetheless supported H6 and H7. Similarly, SE substantially affected both PEOU (*β* = 0.392, *p* < 0.001) and PU (*β* = 0.340, *p* < 0.001), lending support to H8 and H9. This suggests that students who exhibit greater self-efficacy and multimodal literacy are predisposed to regard AI tools as simple to use and helpful, thus reinforcing the role of digital competencies in AI adoption.

Moreover, university support (US) proved to enhance both PEOU (*β* = 0.288, *p* < 0.001) and PU (β = 0.179, *p* < 0.001), thereby supporting H10 and H11. These results highlight that institutional support significantly influences students’ attitudes toward AI tools, particularly regarding the tools’ user-friendliness and perceived value.

The structural model’s explanatory and predictive power was assessed through the coefficient of determination (*R*^2^), effect size (*f*^2^), and predictive relevance (*Q*^2^), as presented in Table 9. The data suggest that the model contributed 47.8% to the explained variance in Attitude (*R*^2^ = 0.478), 59.5% in Intention (*R*^2^ = 0.595), 48.2% in PEOU (*R*^2^ = 0.482), and 38.6% in PU (*R*^2^ = 0.386), therefore demonstrating moderate to substantial explanatory power (Chin, 2010; Hair et al., 2012). Effect sizes (*f*^2^) were assessed in accordance with Cohen’s (1988) guidelines, wherein values of 0.02, 0.15, and 0.35 are construed as small, medium, and large, respectively. The statistical results affirmed that all of the exogenous variables had adequate influence on their respective endogenous constructs. Furthermore, all endogenous constructs showed *Q*^2^ values greater than zero (ATT = 0.362, IN = 0.429, PEOU = 0.348, PU = 0.269), thus confirming the model’s good predictive relevance (Fornell and Larcker, 1981).

**Table 9:** *R*^2^, *Q*^2^, and *f*^2^ Values.

Endogenous variables	*R* ^2^	*Q* ^2^	Structural path	*f* ^2^
ATT	0.478	0.362	PEOU → ATT	0.033
			PU → ATT	0.281
IN	0.595	0.429	ATT → IN	0.223
			PEOU → IN	0.037
			PU → IN	0.073
PEOU	0.482	0.348	ML → PEOU	0.021
			SE → PEOU	0.151
			US → PEOU	0.119
PU	0.386	0.269	ML → PU	0.036
			SE → PU	0.096
			US → PU	0.039

To examine potential mediation effects, bootstrapping was performed following the model specifications provided by Hair et al. (2017), and the findings are summarized in Table 10. It was revealed that SE, US, and ML each exerted significant indirect effects on attitude (ATT), mediated by both PU and PEOU. All of the indirect effects proved significant (*p* < 0.05), thus affirming PU and PEOU as mediators in the formation of users’ attitude toward AI tools. Because the link from attitude (ATT) toward AI to intention (IN) to use it has been extensively validated in prior theory, only the mediation effects leading to ATT are reported here.

**Table 10:** Analysis of mediating effects.

Mediation	Mediator	Indirect effect	Total effect	*P*-value
SE → ATT	PU	0.186	0.259	0.000
	PEOU	0.073		0.003
US → ATT	PU	0.098	0.151	0.000
	PEOU	0.053		0.001
ML→ATT	PU	0.117	0.146	0.004
	PEOU	0.028		0.022

### 4.5 Open-ended question insights

The questionnaire concluded with two open-ended questions: “What is your view on the role of AI tools in enhancing your academic experience? What challenges or limitations do you think may arise from using these tools?”

Many participants provided detailed responses, some of which are presented in Table 11. As can be seen in Table 11, after those statements had been filtered and coded, the majority were found to support most of the proposed hypotheses. These qualitative data offer additional validation of the hypotheses, from a different perspective.

**Table 11:** Hypotheses, coding, and example statements from the final open-ended questions.

Support hypotheses	Coding	Example statements about the future of AI
H2	Perceived usefulness, students’ AI attitudes	It has a positive role, but I am not proficient in the use of AI tools and cannot fully apply them to assist learning and work.
		AI tools have played a very positive role in improving my academic experience. In the face of the needs of modern advanced social productivity, we must be able to master AI proficiently and use AI to actively assist the production and life of human society. At the same time, AI may be challenging to human emotional communication and emotional cognition.
H4, H5	Perceived ease of use, perceived usefulness, Students’ intention to use AI tools	You can expand your knowledge. It can provide ideas for homework, but there may be plagiarism issues. It can systematically improve my views and make supplementary plans. It is more convenient to understand academic knowledge and the information is more comprehensive. Use requires guidance.
		When writing papers and academic reports, I will use AI to help me find and integrate relevant information, saving me time and effort and improving efficiency.
H6, H7	Multimodal literacy, perceived ease of use, perceived usefulness	AI tools can help quickly organize literature, generate preliminary literature reviews, extract key information, etc., greatly saving time in searching and screening materials. AI can recommend relevant literature, databases, and research resources based on research topics to help researchers have a more comprehensive understanding of the latest developments in the field.
		AI tools play an important role in improving the academic experience. They can help students and researchers process data, conduct literature reviews, generate reports, and perform complex calculations more efficiently. For example, AI can help automate data analysis, provide personalized learning suggestions, and even assist in writing papers. These tools can save time, improve work efficiency, and help users better understand complex concepts.
		It can improve efficiency in literature retrieval and reading, academic exchanges and cooperation, and provide many conveniences for academic research.
H7	Multimodal literacy, perceived usefulness	AI tools can help us better understand academic content and help us integrate learning resources.
		AI can recommend learning resources according to personal needs, optimize learning paths, and improve learning effects.
		Improve academic experience and reduce reading comprehension.
		Intelligent writing collaborative system: integrated knowledge retrieval, grammar proofreading, and citation management full process support.
H8	Perceived ease of use, self-efficacy	Convenient and fast.
		More convenient, but too much information still requires independent thinking, independent thinking is still very important.
		AI can improve our learning efficiency and facilitate our information retrieval. However, the authenticity of the information it provides needs to be verified, and some information may not be true and reliable.
H9	Perceived usefulness, self-efficacy	AI tools can help me better obtain and organize academic resources, which will save my time in information collection.
		Reasonable use can help us do better, which may cause certain impacts.
		Will provide some useful information, but it may not be applicable to me.
		We may sometimes rely too much on AI tools, resulting in our lack of independent thinking ability.
		Compared with human thinking, AI is still incomparable. It can help us better solve some academic problems, but we cannot rely on it. It can help us open our minds.
H11	Perceived usefulness, university support	It is very helpful for schools.
		Open your mind and get personalized academic support.

## 5 Discussion and conclusions

### 5.1 Theoretical implications

This study integrated multimodal literacy, self-efficacy, and university support as determinants of AI tool acceptance for university students, drawing upon the technology acceptance model as the theoretical basis. The findings demonstrate that both multimodal literacy and self-efficacy play vital roles in improving students’ perceived ease of use and perceived usefulness of AI-based technologies. This finding aligns with those from earlier studies concerning digital competencies and technology use (Bulut et al., 2015): students who are adept at processing multimodal information and who possess confidence in their abilities are inclined to find AI tools intuitive and beneficial (Compeau and Higgins, 1995; Falebita and Kok, 2024). In addition, the significant role of university support in shaping both the students’ perceived ease of use and their perceived usefulness of AI highlights the critical impact of institutional and environmental factors. Institutional backing—through resources, training, and encouragement—can bridge the gap between personal ability and adoption of technology, underscoring that even capable students benefit from a supportive infrastructure (Sova et al., 2024). The study also confirmed the main TAM relationships regarding the acceptance of AI tools and emphasized that usefulness appears to be a greater determinant of attitude than ease of use is (Almasri, 2024).

### 5.2 Managerial implications

This study suggests practical ways to support AI technologies in academic environments. The first essential step is to develop students’ multimodal literacy. Embedding related skills into coursework can help students better interpret digital content (Davis, 1989; Liang et al., 2023), making AI tools seem more accessible and useful (Ng, 2012). The development of self-efficacy stands as another essential factor. Universities can offer training, peer support, and low-stress environments (Bandura, 1997) in which students feel safe exploring AI tools (Ifenthaler and Schweinbenz, 2013). Finally, universities should establish a robust support system that includes technical assistance, ethical AI workshops, and a modern digital infrastructure to ensure the seamless availability of AI tools for learners (Zawacki-Richter et al., 2019). As Chan and Lo (2025) emphasized, such efforts should also address risks associated with data privacy and algorithmic transparency, because insufficient safeguards in AI deployment may lead to unintended human rights consequences.

## 6 Conclusion

This study examined the key factors that influence students’ adoption of AI tools in higher education and yielded several important insights. First, multimodal literacy, self-efficacy, and university support were all found to positively shape students’ perceptions of AI tools’ ease of use and usefulness. Of the three, self-efficacy and institutional support had relatively stronger effects than multimodal literacy did, highlighting the roles of student confidence and organizational infrastructure in promoting favorable perceptions. Second, perceived ease of use and perceived usefulness were confirmed as important determinants of students’ attitudes toward AI tools, suggesting that their perceptions of functionality and value work in tandem to shape their receptiveness. Finally, students’ behavioral intentions were significantly predicted by the perceived usefulness and ease of use of the AI tools, along with the students’ attitude, with perceived usefulness standing out as the most significant contributor. These findings underscore the fact that although usability and a positive attitude matter, students’ decisions about AI adoption are ultimately anchored in whether they believe AI to be genuinely beneficial to their academic success.

These results also carry important implications for institutions that seek to integrate AI meaningfully into higher education. The findings highlight the value of providing consistent institutional support and of embedding into university curricula digital competencies that will enhance students’ confidence in using technology. At the same time, effective adoption must address the broader ethical and social challenges that accompany increased reliance on AI—such as authorship ambiguity, academic integrity, and the evolving conceptions of student agency (Lo et al., 2025). By proactively responding to these complexities, universities can foster more thoughtful and sustainable engagement with AI while still upholding the core values of higher education in an increasingly digital landscape.

## 7 Limitations and future research

### 7.1 Limitations

The study had several limitations that need to be acknowledged. First, respondents came predominantly from central and eastern China, with limited representation from the western regions, and convenience sampling was employed, thus potentially restricting the representativeness and broader applicability of the findings. Second, the study relied exclusively on self-reported measures, raising concerns about possible social desirability and response biases because some of the participants may have given responses that were influenced by social desirability bias, rather than providing fully accurate reflections of their true standpoints or behaviors. Last, the cross-sectional design limited the study’s capacity to track changes in students’ views or behaviors over time, thereby constraining the ability to infer causal relationships and capture dynamic shifts in attitudes and technology usage.

### 7.2 Future research

Future studies should incorporate more geographically diverse samples, particularly from the underrepresented western regions of China, using probability sampling techniques to enhance generalizability of their findings. Combining self-reported data with objective measures or behavioral tracking could also mitigate potential response and social desirability biases. Future studies may wish to adopt longitudinal or experimental designs to track evolving student perceptions, employ mixed-methods approaches for richer insights, and continuously refine measurement tools to align with the rapid evolution of AI technologies. Finally, future research might specifically investigate the educational impact and effective pedagogical integration of generative AI tools, in order to provide concrete recommendations for educational institutions, policymakers, and curriculum designers.

## Data Availability

The raw data supporting the conclusions of this article will be made available by the authors, without undue reservation.
